# The Hospital Saturday Fund

**Published:** 1902-03-29

**Authors:** 


### THE HOSPITAL SATURDAY FUND.

### THE FIGHT AGAINST SMALL-POX.

THE last two annual meetings of this Fund have differed somewhat in character from their predecessors, and Mr. HAMILTON HOARE summed up the difference rather neatly on Saturday afternoon (22nd inst.) when he said that instead of being merely congratulatory, the meetings had now taken on a more educational aspect. At the meeting early in November, Sir James Crichton Browne lectured on tuberculosis, while on this occasion a lecture was given by Dr. Theodore Dyke-Acland on "Our Fight against Small-pox; or, Common Sense and Vaccination.'

This is not the only innovation. On November 2nd, when the last annual meeting was held, the Chairman, Sir Savile Crossley, intimated that he intended to try and get the date altered, so that the business and accounts should come before the supporters of the Fund within a short time of the winding up for the year, instead of late in the autumn, when there was no particular reason for meeting, and when another financial year was nearly completed. With so much success has the Chairman laid the matter before the executive that it was decided to hold the meeting, as other business meetings are held, early in the year, and the date was fixed for Saturday, the 22nd. If any fears had been entertained that the alteration would have the effect of reducing the number attending the meeting, they must have been completely dispelled on Saturday afternoon, for the large Egyptian Hall at the Mansion House was even fuller than on the occasion of the meeting in November alluded to above. The working classes predominated, men and women being present in about an equal proportion; a detachment of the ambulance service was conspicuous in uniform at the top of the hall; and on the platform were the members of the Council, with Sir Saville Crossley, Sir M. M. Bhownaggree, M.P., Mr. Hamilton Hoare, Dr. Lister, the lecturer, the ladies attached to the ambulance department, and other supporters of the Fund.

At four o'clock Alderman Treloar was announced, and took his place in the chair, as a substitute, he explained later, for the Lord Mayor, who was taking a few days' rest at the seaside. The Chairman's remarks were brief: he said that the Hospital Saturday Fund was an organisation that should appeal to everyone; it was formed by working men for the benefit of the working classes, men, women, and children, and its principle was to help those who were willing to help themselves. It had never departed from its original ideals. After a brief allusion to the subject of the lecture the Chairman asked Sir Saville Crossley to make a statement about the Fund.

### Progress all Along the Line.

Sir Saville Crossley said he was extremely glad to see such a large number present, not only because they were all supporters of the Fund, but because he was extremely anxious that as many people in London as possible should hear the lecture by Dr. Dyke-Acland, who, he felt, was conferring a great compliment on the Fund. The report of the Council would shortly be in the hands of the members, and they would see in it what very good work had been done during the past twelve months. Alluding to the short interval that had elapsed between this meeting and the last annual meeting, the speaker said he was to a great extent responsible for the alteration of date. He thought the alteration was one that would commend itself to all business men. What they wanted was to do their business at a time when the accounts were completed, and not when the facts and figures for the next year were not ready, and there was no particular reason for meeting. There was a distinct reason for meeting that day. It was most gratifying to him to be able to say that the results of last year's work formed a record. The contributions to the income were £21,547, being an increase of £1,266 on those for 1900, and £1,049 more than those for 1897, when the last annual street collection was made. He thought this showed very steady progress, and he was especially glad that this Fund should progress, as the other kindred funds were doing. He was sure that these three funds had been very conducive to the improvements that had been made in the hospitals of London during the last few years. With regard to the distribution, he could assure them they need not desire a more satisfactory balance-sheet. The various standing committees had been formed with the greatest possible care, and the gentlemen appointed to some of them had attended the meetings most regularly and had performed their duties most efficiently.

### The Fund and Tuberculosis.

During the past few years, the speaker went on, a great light had been thrown on the numbers who were suffering from consumption, and it had been found that many patients could be cured both more easily and more cheaply than was even imagined possible. The Fund had been instrumental in helping to cure some for whom otherwise recovery would have been impossible. Through arrangements by which the Fund and the patient shared the cost, no fewer than 71 sufferers from tuberculosis of the organs of respiration were accommodated in hospitals and convalescent homes; these were hopeful cases, who would, however, have stood little or no chance otherwise in the crowd awaiting treatment.

### Harmony among Officials.

As Chairman, he wished to point out the great harmony that had prevailed at all the meetings. A very large number had been held, and although there was frequently a difference of views, yet he was very much indebted to the officials for making his post as Chairman such an easy one to fill. He had much pleasure in moving: "That this meeting cordially approves the efforts of the Hospital Saturday Fund to interest the working classes in the work of the medical charities of London, and also to provide opportunities by which they can contribute, according to their means, towards the support of those institutions."

### "Twice Blessed."

Sir M. M. Bhownaggree, in seconding the resolution, said he felt the most cordial sympathy for the work of the fund. He had had many opportunities of observing the distress to which the working classes were exposed, and he recognised the valuable work the Fund was doing. One of its many features that appealed to him was that of co-operation. He thought it was now generally recognised throughout the civilised world that charity which partook of the nature of co-operation was, like mercy, twice blessed. Based upon that spirit, the Fund promoted the principle of self-help; and through the combined efforts of the self-respecting poor, who wished to contribute to the best of their medical treatment, and the distributions made by the Fund, it was possible to meet the necessities of suffering and distress.

The presentation of certificates, and of medals, for the final examination in ambulance work, was then made by Alderman Treloar; the latter, he said, were very pretty, and not unlike the Victoria Cross. During the rather slow progress of this ceremony, which would have been hastened if the recipients had been collected at the top of the room, instead of having to make their way from all quarters, there was a good share of applause, and at its conclusion the Chairman asked Dr. Theodore Dyke-Acland to give his lecture.

### The Lecture.

The lecture will be given in full in the next number of *The Hospital Saturday Fund Journal.* It was illustrated with limelight views, tables of statistics, and a page from the parish register of Pudsey for 1787, showing the numerous deaths from small-pox in that year, previous to the introduction of vaccination. The lecture was followed with the closest attention throughout, and at its close Sir Saville Crossley proposed the following resolution:—

> "That in the opinion of this meeting of the supporters of the Hospital Saturday Fund the public interest demands that the Government should undertake the establishment of a laboratory fitted in the most perfect manner and adequate to supply all practitioners throughout the United Kingdom with vaccine lymph; and that they should undertake the inspection of all establishments for the preparation of lymph in this country, and regulate the sale of all that is imported from abroad."

The resolution was declared unanimously carried; it was, however, evident that a good many people refrained from voting. A gentleman who said he stood on the platform as one of the unvaccinated declared himself converted, and announced his intention of "having himself done" after the object-lesson given by Dr. Acland.

Dr. Acland replied, and the meeting terminated.

